# Co-expression Patterns between *ATN1* and *ATXN2* Coincide with Brain Regions Affected in Huntington’s Disease

**DOI:** 10.3389/fnmol.2017.00399

**Published:** 2017-11-30

**Authors:** Arlin Keo, N. Ahmad Aziz, Oleh Dzyubachyk, Jeroen van der Grond, Willeke M. C. van Roon-Mom, Boudewijn P. F. Lelieveldt, Marcel J. T. Reinders, Ahmed Mahfouz

**Affiliations:** ^1^Computational Biology Center, Leiden University Medical Center, Leiden, Netherlands; ^2^Delft Bioinformatics Lab, Department of Intelligent Systems, Delft University of Technology, Delft, Netherlands; ^3^Department of Neurology, Leiden University Medical Center, Leiden, Netherlands; ^4^Department of Radiology, Leiden University Medical Center, Leiden, Netherlands; ^5^Department of Human Genetics, Leiden University Medical Center, Leiden, Netherlands

**Keywords:** gene co-expression, polyglutamine diseases, human brain, Huntington’s disease, neurodegeneration, MRI, spinocerebellar ataxias

## Abstract

Cytosine-adenine-guanine (CAG) repeat expansions in the coding regions of nine polyglutamine (polyQ) genes (*HTT*, *ATXN1*, *ATXN2*, *ATXN3*, *CACNA1A*, *ATXN7*, *ATN1*, *AR*, and *TBP*) are the cause of several neurodegenerative diseases including Huntington’s disease (HD), six different spinocerebellar ataxias (SCAs), dentatorubral-pallidoluysian atrophy, and spinobulbar muscular atrophy. The expanded CAG repeat length in the causative gene is negatively related to the age-at-onset (AAO) of clinical symptoms. In addition to the expanded CAG repeat length in the causative gene, the normal CAG repeats in the other polyQ genes can affect the AAO, suggesting functional interactions between the polyQ genes. However, there is no detailed assessment of the relationships among polyQ genes in pathologically relevant brain regions. We used gene co-expression analysis to study the functional relationships among polyQ genes in different brain regions using the Allen Human Brain Atlas (AHBA), a spatial map of gene expression in the healthy brain. We constructed co-expression networks for seven anatomical brain structures, as well as a region showing a specific pattern of atrophy in HD patients detected by magnetic resonance imaging (MRI) of the brain. In this HD-associated region, we found that *ATN1* and *ATXN2* were co-expressed and shared co-expression partners which were enriched for DNA repair genes. We observed a similar co-expression pattern in the frontal lobe, parietal lobe, and striatum in which this relation was most pronounced. Given that the co-expression patterns for these anatomical structures were similar to those for the HD-associated region, our results suggest that their disruption is likely involved in HD pathology. Moreover, *ATN1* and *ATXN2* also shared many co-expressed genes with *HTT*, the causative gene of HD, across the brain. Although this triangular relationship among these three polyQ genes may also be dysregulated in other polyQ diseases, stronger co-expression patterns between *ATN1* and *ATXN2* observed in the HD-associated region, especially in the striatum, may be more specific to HD.

## Introduction

Polyglutamine (polyQ) diseases are a family of nine neurodegenerative disorders caused by a cytosine-adenine-guanine (CAG) trinucleotide repeat expansion in the coding region of one of the polyQ disease-associated genes. PolyQ diseases include Huntington’s disease (HD), six spinocerebellar ataxias (SCAs) dentatorubral-pallidoluysian atrophy (DRPLA), and spinobulbar muscular atrophy (SBMA), each with its own causative gene: *HTT*, *ATXN1*, *ATXN2*, *ATXN3*, *CACNA1A*, *ATXN7*, *TBP*, *ATN1*, and *AR*, respectively (Williams and Paulson, 2008). With the exception of SBMA, all polyQ diseases are inherited in an autosomal dominant manner (Williams and Paulson, 2008). The CAG repeat region is translated into a stretch of glutamine amino acids, also referred to as the polyQ tract (Van De Warrenburg et al., 2002; Jana et al., 2005). In HD patients, the polyQ expansion in the huntingtin protein causes neurodegeneration that affects the striatum most severely and results in cognitive, psychiatric as well as motor disturbances and gait abnormalities (Rubinsztein, 2002; Paulsen et al., 2014). It is thought that the expansion of the polyQ tract causes the protein to misfold and aggregate, and therefore loses its normal function. Hence, these mutant proteins also become toxic components as they trigger the misfolding of other proteins (Arrasate and Finkbeiner, 2012).

In HD and SCAs, longer CAG repeat lengths in the causative polyQ genes are associated with an earlier age-at-onset (AAO) (Hmida-Ben Brahim et al., 2014; Tezenas et al., 2014). In HD, up to 75% of the variability in AAO can be explained by the *HTT* CAG repeat length (Hmida-Ben Brahim et al., 2014), while in SCA1, SCA2, SCA3, SCA6, and SCA7, the CAG repeat in the causative gene explains between 32 and 80% of the AAO variability (Tezenas et al., 2014). There is also evidence suggesting that, in addition to the expanded CAG repeat length in the causative gene, the normal CAG repeat lengths in other non-causative polyQ genes affect the AAO in HD and SCA’s (Pulst et al., 2005; De Castilhos et al., 2014; Hmida-Ben Brahim et al., 2014; Tezenas et al., 2014; Chen et al., 2016; Stuitje et al., 2017). For example, clinical observations show that the AAO in HD is not only affected by the expanded CAG repeat length in *HTT*, but also by the normal CAG repeat length in *ATN1* and *ATXN1* negatively affecting the AAO (Hmida-Ben Brahim et al., 2014). The observation that the number of CAG repeats in multiple polyQ genes can affect the AAO in HD or SCAs suggests that polyQ gene products are functionally interacting.

In addition to the suggested genetic associations based on shared effects on AAO, similar mechanisms likely contribute to polyQ disease pathogenesis. These mechanisms include misfolding of the disease protein, deleterious protein interactions, transcriptional dysregulation, mitochondrial dysfunction, aberrant neuronal signaling, cellular protein homeostasis impairment, and RNA toxicity (Williams and Paulson, 2008). Despite the similarities, these mechanisms seem to affect specific brain regions depending on the particular polyQ disease. To get a better understanding of how the CAG expansion in *HTT* affects the brain, multiple studies used genome-wide expression analysis of post-mortem samples collected from different brain regions. By comparing the brain region-specific expression profiles of HD patients and healthy controls, the highest number of differentially expressed genes was found in the caudate nucleus and to a lesser extent in the cerebellum (Hodges et al., 2006). This demonstrates that differential gene expression in the HD brain has a regional pattern corresponding to the known pattern of neuropathology. A more recent study used longitudinal RNA-sequencing expression data of HD knock-in mice with increasing CAG repeat lengths, and using weighted gene co-expression network analysis (WGCNA), they studied length-dependence of molecular networks in HD-relevant brain regions (Langfelder et al., 2016). They found one striatal module which showed strong patterns of downregulation that were both CAG length- and age-dependent.

In addition to patient postmortem samples and mouse models, characterizing expression patterns of wild-type genes in the healthy brain can provide useful insights into the molecular and pathological changes regarding polyQ diseases. Using gene expression data from ten different brain tissues and 101 healthy individuals from the UK Brain Expression Consortium (Trabzuni et al., 2011), Bettencourt et al. analyzed the co-expression relationships of SCA genes in the healthy brain using WGCNA (Bettencourt et al., 2014). Two cerebellar modules were enriched in SCA transcripts of which one module seemed preserved across all brain regions and the other module seemed unique to the cerebellum. The study suggests that genes co-expressed with SCA genes across all brain regions give rise to more complex phenotypes (other ataxia syndromes and neurodegenerative disorders), while cerebellum-specific co-expressing genes results in pure ataxia phenotypes. While previous studies have focused on region-specific gene expression differences between patients and healthy controls (Hodges et al., 2006; Neueder and Bates, 2014), and co-expression relationships between SCA genes in the healthy brain (Bettencourt et al., 2014), there is no detailed assessment of the relationships among polyQ genes in pathologically relevant regions of the brain.

**FIGURE 1:**
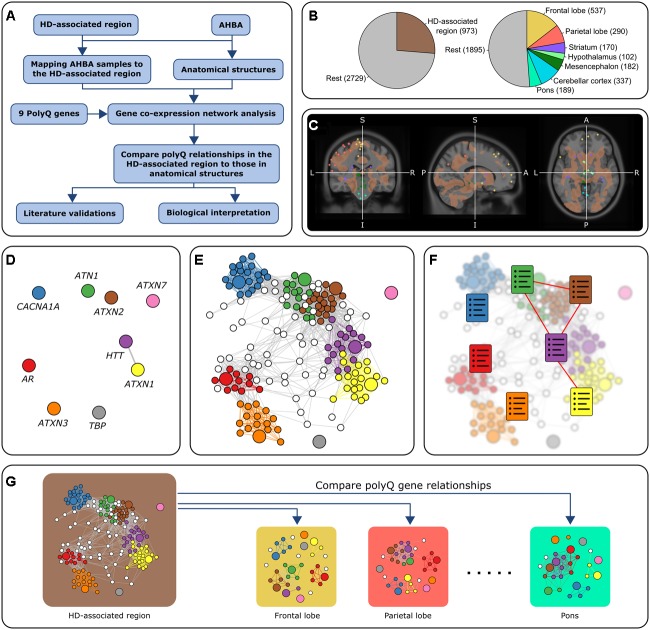
Overview of the approach. **(A)** Flowchart of the general approach in this study using co-expression analysis. **(B)** Number of samples in all six donors. The left chart shows the number of samples in the HD-associated region and the rest of the brain. The right chart shows the number of samples in seven anatomical brain structures and the rest of the brain. There are 3,702 samples in total. **(C)** Mapping AHBA samples to the HD-associated region. AHBA samples were spatially mapped to the HD-associated region (brown) which was based on an MRI study that looked at gray matter changes between HD patients and controls (Coppen et al., 2016). In this MRI image, we discarded all the samples that did not belong to one of the predefined regions. The colored points correspond to samples collected from anatomical structures that fall either inside or outside the HD-associated region. **(D–F)** Co-expression networks for the HD-associated region and each anatomical structure were constructed by retaining links between strongly correlated gene pairs (Pearson’s *r* > 0.5). PolyQ genes are colored and have a larger node size. The relationships between polyQ genes were analyzed based on three methods: **(D)** Direct co-expression between polyQ genes. **(E)** Subnetworks of genes co-expressed with polyQ genes. Nodes are colored according to the co-expressed polyQ gene. **(F)** Functional enrichment of the set of genes co-expressed with each of the polyQ genes (colored charts). Functional links between polyQ genes were assessed based on the overlap of enriched functional terms. **(G)** PolyQ gene relationships in the HD-associated region were then compared to those in networks constructed for different anatomical structures.

We aim to find common mechanisms through which the nine polyQ genes could interact with each other in order to understand how these interactions could affect neuropathology in polyQ disorders. Our approach consisted of interrogating healthy brain gene expression data from the Allen Human Brain Atlas (AHBA) (Hawrylycz et al., 2015) as its high spatial resolution allows localizing interactions to specific brain regions. PolyQ genes with similar expression patterns in a specific brain area suggest their co-involvement in functions specific to that brain area. Using the AHBA to relate biological functions to disease and pinpoint pathways to specific regions of the brain has been reported previously, for example through evaluation of normal activity in the brain of genes associated to migraine (Eising et al., 2016), and autism (Parikshak et al., 2013; Willsey et al., 2013; Mahfouz et al., 2015). Here, we follow a similar approach to detect functional relationships between the nine polyQ genes. We focus on a brain region consisting of several brain areas which were recently shown to be most severely affected in HD through an MRI-guided unbiased approach (Coppen et al., 2016). In addition, the analysis was repeated for seven anatomical brain structures to assess whether the relationships between polyQ genes within the HD-associated region are reflected in other anatomical brain structures (**Figure 1**). We analyzed gene co-expression networks within the different brain regions to identify region-specific relations among polyQ genes and their underlying functional mechanisms.

## Materials and Methods

### Spatial Gene Expression in the Healthy Human Brain

We used the publicly available gene expression data set from AHBA^1^ (Hawrylycz et al., 2015) to exploit possibilities to study neurodegenerative diseases in the healthy brain. The spatially high-resolution data set contains genome-wide microarray expression of 3,702 samples collected from 6 healthy adult donors (5 males and 1 female, mean age 42, range 24–57 years). For each brain, 363-946 samples are available. In two out of the six brains, samples were collected from both hemispheres, while in the remaining four brains, samples were collected from the left hemisphere only.

The expression of several genes in the AHBA was assessed using multiple probes. We selected one probe per gene as follows: (1) if there was one probe for a gene, this probe was selected; (2) if there were two probes for a gene, we selected the probe with the highest variance (measured per brain and then averaged across the six brains); (3) if there were more than two probes for a gene, we chose the probe with the highest connectivity (measured as the sum of the Pearson correlations per brain and then averaged across the six brains). These steps resulted in 19,992 genes selected for further analysis.

### Mapping AHBA Samples to the HD-Associated Region

In our analysis, we included a brain region affected in HD patients compared to healthy controls defined according to gray matter changes in MRI scans (Coppen et al., 2016). Several structural covariance networks (SCNs), corresponding to discontinuous brain areas, were associated with HD. The SCN analysis was applied on whole brain images rather than predefined regions (Minkova et al., 2016) to reveal a map of regions affected in HD. Two disjoint brain areas, referred to as the caudate nucleus and hippocampal network, showed strong significant association with pre-manifest HD and manifest HD patients. The caudate nucleus network includes the nucleus accumbens, pallidum, putamen, and precuneus. The hippocampal network includes the parahippocampal gyrus, cerebellum, pallidum, and planum polare. We combined these two regions into one, hereafter named “HD-associated region.” AHBA samples were mapped to the HD-associated region using the MNI coordinate space. Based on this mapping, we identified AHBA samples located inside the HD-associated region that exhibits significant gray matter volume changes in HD compared to healthy controls.

### Differential Gene Expression in the HD-Associated Region

We examined gene expression differences between samples located inside and outside the HD-associated region. Differential expression was assessed for each brain separately using two one-tailed Mann–Whitney *U*-tests to identify up- and downregulated genes. Genes with a *p*-value lower than 0.025 in either the upper tail or lower tail in five out of six brains were considered differentially expressed.

### Region-Specific Co-expression Analysis

We used Pearson correlation (*r*) as a measure of co-expression between two genes. Gene pairs showing a co-expression greater than 0.5 were considered to be related as they have similar expressions across tissue-specific samples. The co-expression threshold was based on our observation of the polyQ co-expression distributions in different regions (Supplementary Figure 1). For each polyQ gene, a correlation threshold of 0.5 on average coincides with selecting the top 5% most co-expressed genes. Similar patterns of the number of co-expressed genes were observed when the co-expression threshold was set at 0.4 and 0.6 (Supplementary Figure 2). Co-expression was calculated for all possible gene pairs across samples inside the HD-associated region as well as samples representing one of the following brain structures: frontal lobe, parietal lobe, striatum, hypothalamus, mesencephalon, cerebellar cortex, and pons. Co-expression was first calculated per donor and then averaged across all donors to one consensus matrix containing all pairwise gene co-expressions.

### Functional Enrichment of Co-expressed Genes

We assessed enrichment in functional terms for sets of genes using DAVID Bioinformatics resources (Huang et al., 2008, 2009). Enrichment analysis was done for genes differentially expressed in the HD-associated region (up- and downregulated) and sets of genes co-expressed with one of the nine polyQ genes. We selected the following annotation categories: GOTERM_BP_ALL, GOTERM_MF_ALL, GOTERM_CC_ALL. Enrichment analysis was done using Entrez IDs as gene identifiers and all 19,992 genes in the AHBA were added as a background list. Functional terms were selected when the Benjamini-corrected *p*-value was lower than 0.05 and when at least two genes were present in the gene ontology category. To summarize the functional terms enriched in genes differentially expressed between the HD-region and the rest of the brain, we used the clustering function from DAVID and retained only terms with a cluster enrichment score > 2.

### PolyQ Interactions Based on Co-expression

We used three different representations of the relationships between polyQ genes, each highlighting a different way of how polyQ genes could be related. (1) Direct co-expression: co-expression between two polyQ genes indicates a strong interaction based on their spatial expression patterns. (2) Shared co-expression: a significant overlap of the two co-expressed gene sets of two polyQ genes suggests that these two polyQ genes are indirectly related to each other through other genes with which they interact. (3) Functional overlap: if two polyQ genes indirectly interact, the overlap between the functional terms enriched in their corresponding sets of co-expressed genes point out whether the two polyQ genes are also functionally related. We assessed the significance of shared co-expressed genes between two polyQ genes using one-tailed Fisher’s exact test. We considered a functional overlap between two polyQ genes when their respective gene sets share at least 10 enriched functional terms.

### Network Analysis

The polyQ gene network for the HD-associated region was visualized using Gephi (Bastian et al., 2009) and the rgexf^2^ R package. In the network, nodes represents polyQ genes (each with a unique color) as well as genes co-expressed with polyQ genes (each assigned the color of the polyQ gene with which it is co-expressed). The node color is mixed when genes are co-expressed with multiple polyQ genes. Edges are colored by the polyQ gene that they connect to. We used ‘Fruchterman-Reingold’ and ‘label adjust’ options for the layout. The circular co-expression plots for each examined region were visualized using Cytoscape (Shannon, 2003) and the RCy3 R package (Shannon et al., 2013). Each node represents one of the nine polyQ genes and node colors indicate their mean expression across samples specific to the indicated regions per donor and then averaged across the six brains. Expression levels increase from blue to white to red. Edge thickness indicates the co-expression strength between two genes, the number of shared co-expressed genes, or number of shared functional terms between two polyQ gene sets.

### Enrichment of Ubiquitin Ligases and DNA Binding Genes

Genes encoding ubiquitin conjugating enzymes (UBE2) were downloaded from the HUGO Nomenclature Committee (Gray et al., 2015) and includes 41 genes. DNA repair and ubiquitination gene sets, consisting of 125 and 40 genes, respectively, were obtained from the Molecular Signature Database (MSigDB) (Subramanian et al., 2005).

## Results

### *ATXN2* Is Differentially Expressed in the HD-Associated Region

To assess normal gene expression changes in regions affected by HD, we examined the differential expression of genes in AHBA samples split according to being inside or outside the HD-associated region. Among the polyQ genes under study, only *ATXN2* was significantly downregulated in five out of six brains (Supplementary Figure 3). All other polyQ genes did not show consistent expression changes across the six brains. The analysis yielded 2,812 (14.1% of all 19,992 genes) significant genes (*p*-value < 0.025; one-sided Mann-Whitney *U*-test; Bonferroni-corrected). Out of the differentially expressed genes, 711 genes showed a significant higher expression in the HD-associated region (upregulated) and 2,101 genes showed a lower expression (downregulated). The set of downregulated genes was enriched in terms related to cytoplasm, mitochondrial processes, cellular component organization, DNA damage recognition, synapses, autophagy, and metabolic processes (Benjamini-corrected *p* < 0.05; term cluster enrichment score > 2; Supplementary Table 1). These biological functions have been implicated in HD before (Rubinsztein, 2002; Hodges et al., 2006; Seredenina and Luthi-Carter, 2012; Groen and Gillingwater, 2015; Jones et al., 2017). Interestingly, there were no functional terms enriched in the set of upregulated genes. From Supplementary Figure 3 it can also be inferred that *ATXN2* had medium expression both inside and outside the HD-associated region in all six brains. Compared to all polyQ genes, *ATN1* had the highest expression across the brain, while *ATXN3* had the lowest expression.

### *ATN1*, *ATXN2* and *HTT* Have the Highest Connectivity among polyQ Genes

We examined the functional relations among the polyQ genes by analyzing their spatial co-expression using the high-resolution AHBA. Co-expression of the polyQ genes was analyzed in the healthy brain using the AHBA samples within regions associated with gray matter changes in HD patients (HD-associated region). In addition, we analyzed seven anatomical brain structures to assess whether polyQ gene co-expression patterns in the HD-associated region are reflected within these structures. All anatomical structures had a sufficient number of samples to perform co-expression analysis (**Table 1**).

**Table 1:** Number of samples for the HD-associated region and seven anatomical brain structures across donors.

Donor	HD-associated region	Frontal lobe	Parietal lobe	Striatum	Hypothalamus	Mesencephalon	Cerebellar cortex	Pons
9861	280	161	81	48	9	48	41	52
10021	183	135	57	46	22	61	76	50
12876	99	47	37	16	17	9	40	6
14380	139	70	42	24	20	22	44	26
15496	125	64	32	18	16	24	60	29
15697	147	60	41	18	18	18	76	26
Total samples	973	537	290	170	102	182	337	189

The HD-associated region overlaps with parts of different anatomical structures. None of the examined anatomical structures were overrepresented in the HD-associated region (Supplementary Table 2). We examined the cerebellar cortex instead of the cerebellum because of strong expression differences between the cerebellar cortex and cerebellar nuclei samples (Huisman et al., 2017). The cerebellar nuclei were left out of the co-expression analysis as they had too few samples. Since the HD-associated region showed little overlap of samples with anatomical structures, co-expression patterns in this region are not dominated by samples from any specific anatomical structures.

**FIGURE 2:**
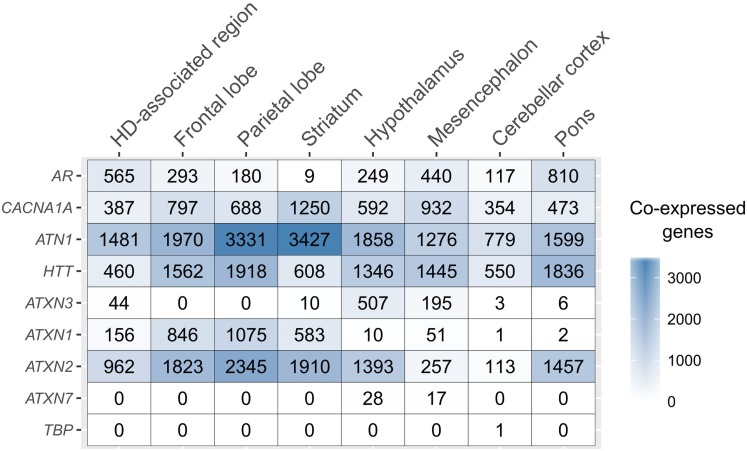
PolyQ co-expressed gene sets across the brain. The number of genes co-expressed with each of the polyQ genes (rows) for the different, anatomical brain structures (columns).

The number of co-expressed genes varied per polyQ gene and brain region (**Figure 2**). *ATN1* had the highest number of co-expressed genes in all examined regions except the mesencephalon and pons where only *HTT* had more co-expressed genes. The largest number of genes co-expressed with *ATN1* was observed in the striatum (3,428 genes), followed by the parietal lobe (3,332), and the frontal lobe (1,971). *ATXN2* had the second largest number of co-expressed genes in the parietal lobe, followed by the striatum and the frontal lobe. *TBP* had only one gene co-expressing in the cerebellar cortex. For all sets of genes co-expressed with polyQ genes we performed functional enrichment analysis to obtain sets of functional GO terms (Benjamini-corrected *p* < 0.05). This was repeated for all inspected brain regions (Supplementary Figure 4). Similar patterns were observed for the number of functional terms and their respective gene set sizes, with *ATN1*, *ATXN2*, and *HTT* showing the highest number of GO terms.

### Strong Connectivity between *ATN1* and *ATXN2* in the HD-Associated Region

**FIGURE 3:**
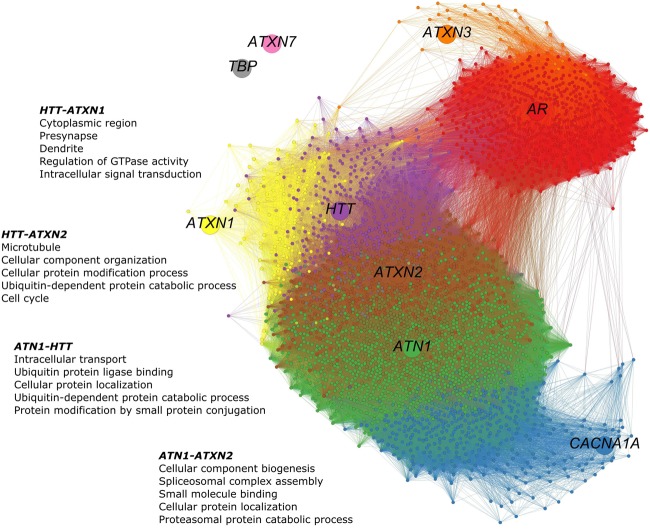
PolyQ gene co-expression network in the HD-associated region. The network consists of 3,368 nodes representing genes. The poly genes are shown as larger nodes, each with a unique color. Genes were considered co-expressed when the calculated Pearson’s correlations exceed 0.5. Smaller nodes are genes that are co-expressed with at least one of the nine polyQ genes. Their colors indicate the polyQ gene with which they are co-expressed. Genes co-expressed with multiple polyQ genes have a mixed color. Edges are colored according to the colors of the nodes they connect. In the HD-associated region, *ATN1*-*ATXN2* is the only gene pair that is directly co-expressed, while other polyQ genes are more distantly related through indirect relationships. In this network, 41 genes co-expressed with *HTT*, *ATN1*, and *ATXN2* and 488 co-expressed with *ATN1* and *ATXN2*.

**FIGURE 4:**
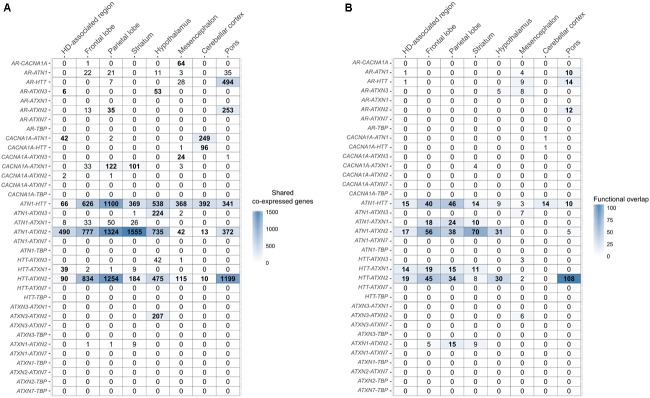
Overlap of co-expressed gene sets and functional terms between polyQ genes across the brain. **(A)** Number of shared co-expressed genes between each polyQ gene pair in different brain regions. Significant overlap of co-expressed genes is indicated in bold numbers (Fisher’s exact test; *p* < 0.05). The respective genes co-expressed with two polyQ genes in the HD-associated region are given in Supplementary Table 3. **(B)** Functional overlap between each polyQ gene pair in different brain regions measured as the number of shared enriched functional terms between their corresponding sets of co-expressed genes. Functional overlaps of at least 10 functional terms are indicated in bold.

The co-expression network of polyQ genes within the HD-associated region includes all genes that are co-expressed with one of the nine polyQ genes (**Figure 3**). The numbers of shared co-expressed genes for different brain regions are shown in **Figure 4A**, and their respective co-expressed genes in the HD-associated region are listed in Supplementary Table 3. The functional overlap between two polyQ genes (**Figure 4B**) was measured as the number of shared enriched functional terms between their corresponding sets of co-expressed genes (Supplementary Figure 4).

**FIGURE 5:**
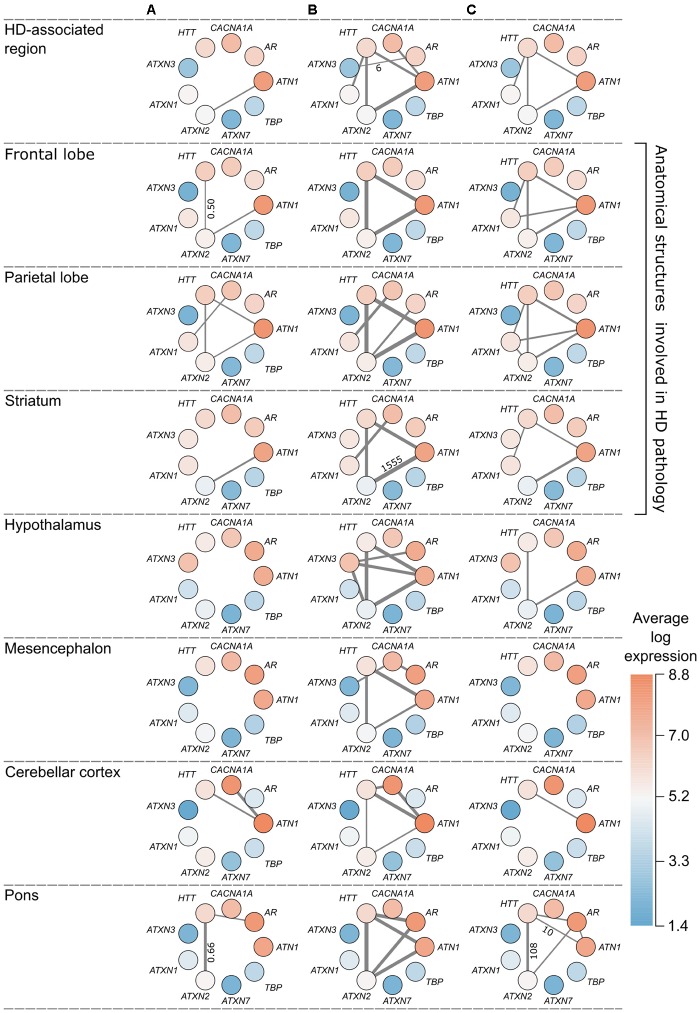
Overview of polyQ relationships in the HD-associated region and seven anatomical structures. Each node represents a polyQ gene and the color represents the expression of that polyQ gene averaged across region-specific samples and the six donors (red indicates high expression and blue low expression). Edge width indicates the strength of the detected relationship between polyQ genes. Each column represents a different type of relationship: **(A)** direct co-expression between polyQ genes, edge width corresponds to the co-expression value; **(B)** shared co-expression, edge width indicates the overlap of co-expressed genes, only for significant overlaps (*p* < 0.05); **(C)** functional overlap, edge width indicates the overlap size between two polyQ gene sets shown only for overlaps of at least 10 enriched terms. For each type of relationship the lowest and highest value are given along the thinnest and thickest edge.

In the HD-associated region, *ATN1*-*ATXN2* was the only polyQ gene pair that was directly co-expressed (*r* = 0.53, **Figure 5**). The same pair also shared most (490) co-expressed genes among all polyQ gene pairs. *TBP* and *ATXN7* were not co-expressed with other genes at all. While both genes show low mean expression levels in the examined regions, the variance is similar to other polyQ genes (Supplementary Figure 5). Their low expression levels indicate that *TBP* and *ATXN7* are less active in these brain regions, suggesting that they are not functionally related to other polyQ genes, at least in the examined regions. The *AR* and *ATXN3* gene sets showed more indirect and distant co-expressions with other polyQ genes. *HTT* shared many co-expressed genes with *ATN1*, *ATXN2*, and *ATXN1* (**Figure 4A**).

### PolyQ Co-expression Relationships Overlap between the HD-Associated Region and Anatomical Structures Involved in HD-Pathology

Co-expression relationships of polyQ genes in the HD-associated region were also observed in other anatomical structures. The direct co-expression between *ATN1* and *ATXN2* in the HD-associated region was also observed in the frontal lobe (*r* = 0.53), parietal lobe (*r* = 0.52), as well as the striatum (*r* = 0.56) that showed the highest correlation (**Figure 5A**). In the striatum, *ATN1*-*ATXN2* was also the only co-expressed polyQ gene pair similar to the HD-associated region. *ATN1* and *ATXN2* had the highest correlations in the striatum and they also had the highest number of overlapping co-expressed genes and functional terms in the striatum. This indicates that the co-expression patterns between *ATN1* and *ATXN2* found in the HD-associated region are particularly pronounced in the striatum.

There is a significant overlap between sets of co-expressed genes of *HTT*, *ATN1*, and *ATXN2* across all studied regions (**Figures 4A**, **5B**). When considering the overlap between sets of enriched functional GO terms (**Figures 4B**, **5C**), the triangular relationship between these three genes is only observed in the frontal and parietal lobes of the cerebral cortex. In the striatum, we observe a very strong functional overlap between *ATN1* and *ATXN2*. Together, these observations suggest a strong regional and functional relatedness between the three genes rather than just the measured co-expression.

In addition, *ATXN1* shows significant overlap of functional terms with *HTT* in the HD-associated region, frontal lobe, parietal lobe, and striatum. *ATXN1* was also found to share functional terms with *ATN1*, *ATXN2*, and *HTT*, forming a clique of four polyQ genes in the parietal lobe. Finally, the co-expression patterns in the HD-associated region seemed to be dominated by the co-expression patterns within the frontal lobe, parietal lobe, and striatum, which were also the regions described as part of the HD-associated region in the imaging study (Supplementary Figure 6; Coppen et al., 2016).

### *HTT*, *ATN1*, and *ATXN2* Are Associated Through Binding, Localization, and Regulation of Cellular Components

To gain insight into the functional relationships between *HTT*, *ATN1*, and *ATXN2*, we examined the overlap of functional terms between all three genes (Supplementary Table 4). These three genes share 15 functional terms, in the frontal and the parietal lobe, showing that they are involved in binding of cellular components. In the striatum, they are involved in the positive regulation of catalytic activity and molecular function, while in the hypothalamus, they are involved in DNA repair and cell cycle process and regulation. In the pons, they are involved in the cytoskeleton and regulation of cellular component organization. In the HD-associated region, they are involved in ubiquitin protein ligase binding, microtubule cytoskeleton, and cellular protein and macromolecule localization. In the mesencephalon and cerebellar cortex *HTT*, *ATN1*, and *ATXN2* do not share functional terms together. Together these overlapping functions between *HTT*, *ATN1*, and *ATXN2* suggest that their connectivity in the aforementioned brain regions is involved in the binding, localization, and regulation of cellular components.

We focused on genes involved in ubiquitination or DNA repair pathways, because these pathways have been previously associated with polyQ diseases (Bettencourt et al., 2014, 2016; Chen et al., 2017). In the HD-associated region, eight *UBE2* genes that are part of the ubiquitin conjugating enzymes gene family, *UBE2G2*, *UBE2I*, *UBE2C*, *CDC34*, *UBE2W*, *UBE2Z*, *UBE2E3*, and *UBE2D4*, co-expressed with polyQ genes. Furthermore, several polyQ genes co-expressed with DNA repair genes, especially *HTT*, *ATN1*, and *ATXN2*. Most DNA repair and ubiquitination genes were co-expressed with *ATN1* in the striatum (34 and 11 genes, respectively; Supplementary Figure 7). In summary, ubiquitin-related genes and DNA repair genes co-expressed with highly connected polyQ genes *HTT*, *ATN1*, and *ATXN2* in regions associated with HD.

## Discussion

Polyglutamine diseases share a similar genetic basis and their phenotypes, such as AAO, are affected by CAG repeat variations in the normal range in polyQ genes other than the causative gene (Pulst et al., 2005; De Castilhos et al., 2014; Hmida-Ben Brahim et al., 2014; Tezenas et al., 2014; Chen et al., 2016; Stuitje et al., 2017). The causative polyQ genes are thought to modulate protein interactions through the role of CAG tracts in stabilizing protein interactions (Schaefer et al., 2012). We analyzed the relationships between nine polyQ genes based on their co-expression within different regions of the healthy human brain.

We performed co-expression analysis in a brain region previously associated with HD specific damage in the cortical and subcortical gray matter. By combining imaging data from HD mutation carriers with spatial gene expression patterns from healthy brains, we were able to study the role of interactions between polyQ genes in the neuropathology of HD. Seven anatomical brain structures were also included in the analysis to examine regional differences compared to the HD-associated region. The anatomical structures also included the striatum, the main affected structure in HD. However, co-expression patterns in the striatum may be more specific to the structure itself rather than to HD pathology.

Among the nine polyQ genes, the relationship between *ATN1* and *ATXN2* was most pronounced based on our three representations of gene associations: it was the only gene pair that directly co-expressed, shared the most co-expressed genes, and also showed functional overlap in the HD-associated region. The same polyQ gene pair also shared co-expressed genes, and a functional overlap with *HTT*, suggesting functional connectivity with the causative gene of HD. Furthermore, out of the nine polyQ genes, only *ATXN2* had a significant lower expression in the HD-associated region compared to the rest of the brain. An expanded polyQ tract in the huntingtin protein may dysregulate pathways in which all three polyQ genes are involved. The function of *ATXN1* may also be affected in HD as this gene also shared co-expressed genes and showed functional overlap with *HTT* in the HD-associated region.

The co-expression patterns among polyQ genes within the HD-associated region overlapped with those observed within anatomical structures involved in HD neuropathology. In the striatum, the main structure affected in HD, *ATN1* and *ATXN2* were directly co-expressed and shared co-expressed genes. This suggests that this gene relationship has a more important role in HD than in other polyQ diseases. For the frontal and parietal lobes, *ATN1* and *ATXN2* were directly co-expressed and shared functional terms together with *HTT*, similar as for the patterns observed for the HD-associated region. The frontal lobe includes the primary motor cortex BA4, and is located next to the parietal lobe, which includes other motor areas. The motor cortex, involved in planning, control, and execution of voluntary movement, has been previously implicated in HD (Pfurtscheller and Berghold, 1989; Fogassi and Luppino, 2005; Nachev et al., 2008; Stinear et al., 2009). The motor cortex is also known to be directly connected to the caudate nucleus of the striatum (Muhammad et al., 2006). In a previous study, HD gene expression profiles in the caudate nucleus and motor cortex were strikingly similar (Hodges et al., 2006), suggesting that similar molecular mechanisms in different brain regions are involved in neurodegeneration. These mechanisms may include polyQ genes as we find similar co-expression patterns in the frontal lobe and striatum between *HTT*, *ATN1*, *ATXN2*, and *ATXN1* (**Figure 5**).

There was a significant overlap in co-expressed genes between *HTT*, *ATN1*, and *ATXN2* in all examined brain regions. It would be interesting to evaluate whether this triangular relation between *HTT*, *ATN1*, and *ATXN2* is dysregulated in all polyQ diseases, especially HD, DRPLA, and SCA2. In addition, we observed strong connectivity of *ATXN2* and *AR* with *HTT* in the pons that has been described to undergo atrophy in SCA2 (Ying et al., 2006). This relationship may possibly be disrupted in the pons of SCA2 patients. Altogether, these region-specific interactions demonstrate that co-expression analysis can reveal many interesting relations between genes based on their spatial information.

Accumulating evidence suggests that normal CAG repeat size variations in polyQ genes could act as genetic modifiers of AAO in different polyQ diseases. For example, the normal CAG repeat length in *ATXN3*, known to have a deubiquitinating function (Matos et al., 2011), has been found to have a positive effect on HD progression as it was associated with a later AAO (Stuitje et al., 2017). There is also evidence for a polyQ-length dependent interference of both the mutant and normal proteins with a range of other protein binding partners (Kaltenbach et al., 2007; Li et al., 2007; Benn et al., 2008; Lim et al., 2008; Yokoshi et al., 2014; Ashkenazi et al., 2017; Becker et al., 2017). Mutant proteins can interact either more strongly or weakly with other polyQ containing proteins and thereby inhibit there physiological function (Ashkenazi et al., 2017; Becker et al., 2017). A case in this regard is a recent study which showed that the polyQ domain of wild-type ataxin 3 enables it to interact with beclin 1, a key initiator of autophagy, allowing the deubiquitinase activity of ataxin 3 to protect beclin 1 from proteasome-mediated degradation and thereby enabling autophagy (Ashkenazi et al., 2017). They demonstrated that mutant huntingtin polyQ fragments competed with ataxin 3 for interaction with beclin 1 in a polyQ-length dependent manner, thereby inhibiting autophagy and contributing to neuronal dysfunction. This finding may explain the protective role of larger *ATXN3* CAG repeat sizes in HD (Stuitje et al., 2017). In our analysis we did not find any relation in co-expression between *HTT* and *ATXN3* in the examined regions. This might be due to the polyQ length-dependent competing between huntingtin and ataxin 3 being relevant only with pathologically expanded polyQ tracts, which were not present in AHBA. Furthermore, atrophin 1 and the androgen receptor have been found to bind to beclin 1 in a polyQ length-dependent way. Among the polyQ genes and examined brain regions in our study, we found that *BECN1* is only co-expressed with *ATN1* in the pons. Several putative interactions with *HTT* reported in previous studies were also supported by the co-expression analysis in our study. In the HD-associated region, *ATN1* and *ATXN2* shared co-expression partners with *HTT* and both genes were also functionally related to *HTT*. For each of the genes it was shown before that variations in the normal CAG repeat length, together with the expanded CAG repeat in *HTT*, affect the AAO in HD (Hmida-Ben Brahim et al., 2014). The co-expression patterns between *HTT*, *ATN1*, and *ATXN1* suggest a functional relationship in brain regions that are involved in HD pathology.

Ubiquitin may be involved in HD pathogenesis through the relationships between *HTT*, *ATN1*, and *ATXN2*. The ubiquitin-proteasome system (UPS) has been linked extensively to the pathogenesis of neurodegenerative diseases, including HD and other SCAs (Williams and Paulson, 2008; Arrasate and Finkbeiner, 2012; Atkin and Paulson, 2014; Dantuma and Bott, 2014; Ortega and Lucas, 2014; Bettencourt et al., 2016). Aggregates in polyQ diseases show parts of ubiquitin and several important homeostatic proteins (Bowman et al., 2005). We found that E3 ubiquitin ligase genes *UBR4* and *CHIP/STUB1*, and ubiquitin-activating *UBA1* gene to be co-expressed with *HTT*, *ATN1*, and *ATXN2*. This suggests that the three polyQ genes contribute to the UPS in the HD-associated region of the healthy brain. Biochemical properties of *CHIP* are well-studied and this gene is known to interact with mutant polyQ proteins (including *HTT*, *ATXN1*, *ATXN3*, and *AR*) suppressing polyQ aggregation by promoting proteasomal degradation (Jana et al., 2005; Miller et al., 2005; Adachi et al., 2007; Williams and Paulson, 2008; Kuiper et al., 2017). *CHIP* has also been implicated in several SCAs indicating it is directly involved in disease pathology (Ronnebaum et al., 2014). The gene *UB4R* has a fundamental role in calcium signaling and neuronal survival and may contribute to neurodegenerative conditions (Parsons et al., 2015). This gene has been identified as a candidate locus in early onset episodic ataxia (Conroy et al., 2014). It is not known whether recognition of aberrant proteins by ubiquitin ligases is beneficial or disadvantageous (Chhangani et al., 2013). *UBA1* is an important regulator of cellular protein homeostasis and contributes to the pathogenesis in SBMA and HD (Liu and Pfleger, 2013; Groen and Gillingwater, 2015). Altogether, we found genes involved in the UPS that co-expressed with multiple polyQ genes which have been associated with similar pathologies in neurodegenerative disorders. The UPS is involved in protein homeostasis and may be affected early in HD through strong regional interactions. If there are changes in huntingtin protein function due to polyQ expansion, then the interaction between *ATN1* and *ATXN2* is likely to be affected. The low expression of these genes in certain brain regions could indicate their increased vulnerability to dysregulations of the UPS. We suspect co-expressed polyQ genes to have a role in AAO, as they and their shared co-expression partners seem to be involved in functions that have been associated to polyQ diseases.

There are several issues that limit a one-to-one comparison between the co-expression relationships reported in the present study and phenotype-genotype associations described previously (Pulst et al., 2005; De Castilhos et al., 2014; Hmida-Ben Brahim et al., 2014; Tezenas et al., 2014; Chen et al., 2016; Stuitje et al., 2017). First, we do not expect all phenotype-genotype associations to be explained by changes in co-expression patterns. Second, there is a wide degree of heterogeneity in both the data and methods used in the genetic association studies (Pulst et al., 2005; De Castilhos et al., 2014; Hmida-Ben Brahim et al., 2014; Tezenas et al., 2014; Chen et al., 2016; Stuitje et al., 2017), limiting the possibility to combine their findings. Third, thus far, not all possible pairs of polyQ genes have been tested in the genetic association studies, highlighting the need for more comprehensive genetic association studies in larger cohorts of patients with polyQ disorders.

To validate our findings in other brain gene expression data we need a dataset with a spatial sampling resolution that allows co-expression analysis within substructures of the brain. Although the UK Brain Expression Consortium (Trabzuni et al., 2011) sampled multiple (10) brain regions per healthy individual, the expression within a substructure, e.g., the cerebellum, is still represented by a single sample. Gene expression data of HD patients may be used to observe whether co-expression patterns are altered in disease state. However, existing datasets are particularly rich in the number of individuals they sampled and not brain regions (Hodges et al., 2006). This captures variation across individuals, while in our gene co-expression networks we capture spatial variation within a brain region of interest (e.g., striatum).

## Conclusion

We showed that polyQ genes are co-expressed in the healthy brain and that their relationships are also specific to certain brain regions including a region associated with HD. Our aim was to find co-expression patterns between polyQ genes in different brain regions. The co-expression networks are likely altered in polyQ diseases due to interaction changes, especially in brain regions associated with the disease. The fact that these findings could not be validated on other expression datasets implies the importance of follow-up studies to understand more about the mechanisms behind polyQ diseases. We show that gene expression in the healthy brain may render specific regions vulnerable to expression changes based on gene co-expression networks.

## Author Contributions

AK, NA, MR, and AM designed the study. BL provided funding and participated in the conceptualization of the study. JvdG provided the imaging data, which was processed by OD. AK performed the data analysis. AK, NA, WvR-M, MR, and AM interpreted the data and wrote the manuscript with input from all authors. AM and MR supervised the overall project. The final manuscript was read and approved by all authors.

## Conflict of Interest Statement

The authors declare that the research was conducted in the absence of any commercial or financial relationships that could be construed as a potential conflict of interest.

## Footnotes

1 http://human.brain-map.org/

2 https://CRAN.R-project.org/package=rgexf
